# “Switch-Off” of Respiratory Sinus Arrhythmia Can Occur in a Minority of Subjects During Functional Magnetic Resonance Imaging (fMRI)

**DOI:** 10.3389/fphys.2018.01688

**Published:** 2018-11-27

**Authors:** Beate Rassler, Andreas Schwerdtfeger, Christoph Stefan Aigner, Gert Pfurtscheller

**Affiliations:** ^1^Carl-Ludwig-Institute of Physiology, University of Leipzig, Leipzig, Germany; ^2^Department of Psychology, University of Graz, Graz, Austria; ^3^BioTechMed-Graz, Graz, Austria; ^4^Institute of Medical Engineering, Graz University of Technology, Graz, Austria; ^5^Institute of Neural Engineering, Graz University of Technology, Graz, Austria

**Keywords:** respiratory sinus arrhythmia, heart rate variability, ∼0.1 Hz oscillations, state anxiety, functional magnetic resonance imaging, emotion regulation

## Abstract

A group of 23 healthy scanner naïve participants of a functional magnetic resonance imaging (fMRI) study with increased state anxiety exhibited 0.1 Hz oscillations in blood-oxygenation-level-dependent (BOLD) signals, heart rate (HR) beat-to-beat intervals (RRI) and respiration. The goal of the present paper is to explore slow oscillations in respiration and RRI and their phase-coupling by applying the dynamic “wave-by-wave” analysis. Five participants with either high or moderate levels of fMRI-related anxiety (age 23.8 ± 3.3y) were found with at least one bulk of consecutive breathing waves with a respiration rate between 6 to 9 breaths/min in a 5-min resting state. The following results were obtained: (i) Breathing oscillations with dominant frequencies at 0.1 Hz and 0.15 Hz displayed a 1:1 coupling with RRI. (ii) Inspiration time was significantly longer than expiration time. (iii) RRI minima (start of HR decrease) coincided with the early inspiration, and RRI maxima (start of HR increase) coincided with the late inspiration. (iv) RRI rhythm led over the respiratory rhythm. This phase-coupling pattern is quite contrary to typical respiratory sinus arrhythmia where HR increases during inspiration and decreases during expiration.

## Introduction

Anxiety as basic emotion has a strong impact on respiration (Homma and Masaoka, 2008). While the general feeling of anxiety typically increases respiratory frequency (Kato et al., 2017), participation in fMRI scanning can induce different breathing patterns including slow breathing at about 0.1 Hz (breathing rate 6/min; Pfurtscheller et al., 2018). fMRI scanning is a basically uncomfortable claustrophobic situation that is usually associated with increased state anxiety (Munn et al., 2015). Both increase of respiration frequency and slow breathing contribute to an enhanced HRV. Such a high HRV is not only associated with a successful regulation of emotions but also represents a type of important resource to process unpleasant emotions like anxiety and stress (Thayer and Lane, 2009). It has been reported that slow forced (e.g., metronome-paced) breathing close to the resonance frequency of the baroreflex loop at 6/min does not only exhibit a high HRV but can also induce emotional well-being (Lehrer, 2013; Mather and Thayer, 2018). In contrast to conscious forced breathing at 6/min, the slow spontaneous breathing during scanning is an autonomic process.

Slow oscillations with a dominant frequency at 0.1 Hz of vascular and neural origin were frequently found in fMRI BOLD signals in resting states. While the phase coupling between BOLD data and RRI time courses in the 0.07–0.13 Hz band was studied in different brain regions, the respiration was not analyzed (Pfurtscheller et al., 2017, 2018). The goal of the present paper is to explore for the first time slow oscillations in respiration and RRI and their phase-coupling by applying the dynamic “wave-by-wave” analysis (Raßler and Kohl, 1996) in healthy participants of a fMRI study.

## Materials and Methods

Twenty-three healthy MRI-naïve people participated in a fMRI study with recording of HR, respiration and evaluation of the state anxiety (Pfurtscheller et al., 2017, 2018). All participants gave informed written consent to the protocol of the study, which had been approved by the local Ethics Committee at the University of Graz. For further details like, experimental paradigm, evaluation of AS (STADI; Laux et al., 2013) within the scanner, electrocardiogram and respiration recording see Pfurtscheller et al. (2018). A visual check of all pairs of respiration and RRI signals in 5 min of resting state recordings (23 participants, four resting states; Pfurtscheller et al., 2018) revealed bulks of consecutive breathing waves with a respiration rate between 6 and 9 breaths/min (0.1 Hz to 0.15 Hz) in 5 participants (age 23.8 ± 3.3 years). In all other participants no bulks of slow breathing waves were observed.

### Wave-by-Wave Analysis of RRI and Respiratory Data

**FIGURE 1:**
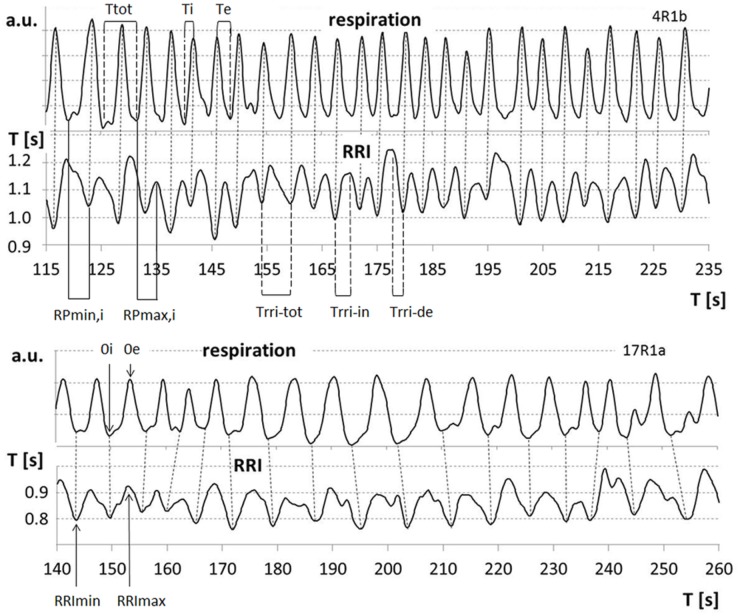
Examples for respiratory sinus arrhythmia (RSA) (upper part), cessation of RSA (lower part) and relevant parameters: 0i/0e: start of inspiration/expiration; Ttot, breath duration; Ti/Te, inspiration/expiration time; RRImin/RRImax, minima/maxima of RRI curve; Trri-tot, duration of RRI wave; Trri-in/Trri-de, increasing/decreasing flank of RRI waves; RPmin,i/RPmax,i, relative phase of start of inspiration and RRI minima/maxima related to Ti.

The duration of breathing and RRI waves for the group of 5 participants showing slow oscillations was detected period by period. Oscillations smaller than 20% of the subject’s average amplitude (arbitrary threshold) were not counted as true periods. Inspiration time was determined as time interval from a minimum in the respiration curve (0i) to the subsequent maximum (0e); Te is the time interval from 0e to 0i; total breath duration (Ttot) is calculated as the sum of Ti and related Te. Analogously, Trri-tot is the time interval from a minimum in the RRI curve (RRImin) to the next one. Trri-tot is divided into an increasing (Trri-in, from RRImin to the subsequent RRI maximum, RRImax) and a decreasing half-period (Trri-de, from RRImax to the next RRImin; Figure 1).

The phase-relationship between respiration and RRI was analyzed using the method of sequential RP plots that has been previously applied for detection of motor-respiratory coupling (Raßler et al., 1990; Raßler and Kohl, 1996; Krupnik et al., 2015). RPs are the time intervals between respiratory phase transitions and RRI maxima or minima expressed in percent of the related Ti and Te, respectively (Figure 1). Breath-by-breath plots of RPs allow detection of the preferred phase-relationship between respiration and RRIs. The preferred RPmin and RPmax, respectively, were calculated as means from all sequences of at least two consecutive breaths with stable RPs.

### Statistical Analysis

Statistical analyses were carried out with the software package SigmaPlot Version 13.0 (Systat Software GmbH, Erkrath, Germany) for Windows. First, we compared each individual’s Ti vs. Te and Trri-in vs. Trri-de. A Shapiro–Wilk procedure was used as test for normality. Differences were tested for significance using a paired *t*-test. If the values were not normally distributed, a Wilcoxon paired rank sum test was applied. Further, the average Ttots and Trri-tots and the frequency of changes in period duration were compared across subjects using a paired *t*-test after normality was assured by a Shapiro–Wilk test.

## Results

The AS (possible range of scores: 10–40) was low in three participants (*AS* = 13, 14, and 16) and moderate to high in two participants (*AS* = 21 and 25). Thus, slow breathing waves were present in participants with low and high anxiety score.

### Frequency-Coupling Between Breathing and RRI Waves

**FIGURE 2:**
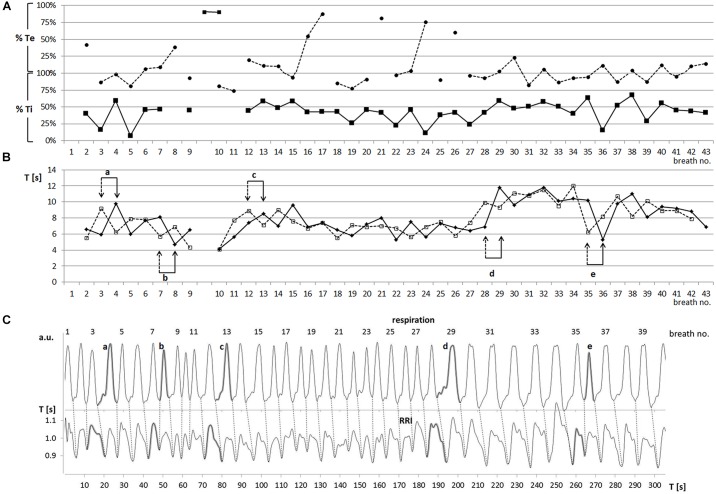
**(A)** Sequential plot of relative phases RPmin (–■–) and RPmax (---●---) [% of Ti and Te] (one characteristic participant: subject 11). Abscissa: breath number; ordinate: RP in % Te (upper part) and % Ti (lower part). **(B)** Sequential plot of duration of breathing (Ttot, –◆–) and RRI (Trri-tot, ---◻---) waves (subject 11). Abscissa, breath number; ordinate, Ttot, Trri-tot [s]. Pairs of arrows (a–e) mark changes in period duration. Note that these changes occur first in the RRI rhythm (dashed arrow) and are followed in the breathing rhythm (solid arrow) one period later. **(C)** Breathing (upper trace) and RRI waves (lower trace; subject 11). Breath number is given above the upper curve. Abscissa, time [s]; ordinate, arbitrary units (upper part), RR interval [s] (lower part). Shaded waves in both curves (a–e) correspond to the paired events a–e in part B.

Two slow rhythms were found. The average breath duration of one rhythm was 6.65 ± 0.33 s corresponding to a breathing rate of 0.15 Hz. RRIs oscillated at the same rate (6.64 ± 0.28 s). However, a more detailed analysis showed that the breathing rate fluctuated over time (Figure 2). Besides this dominant frequency around 0.15 Hz occurring in 45 ± 15% of total recording time, another slow wave rhythm with a frequency around 0.1 Hz (Ttot: 10.04 ± 0.36 s; Trri-tot: 9.93 ± 0.26 s) was observed in 4 out of 5 subjects in 37 ± 10% of the recording time. These breaths and RRI waves were stably coupled at a frequency ratio of 1:1.

Of note, the 5 participants exhibited a breathing pattern with Ti being consistently and significantly longer than Te (Ti 4.0 ± 0.6 s, Te 2.6 ± 0.4 s, *p* = 0.001). The RRI waves showed a similar asymmetry with Trri-de being longer (4.1 ± 0.8 s) than Trri-in (2.9 ± 0.4 s, *p* = 0.01).

### Phase-Coupling Between Breathing and RRI Waves

Of note, Ti was almost equal to Trri-de, and Te was in the same range as Trri-in. This correspondence ensured that the 1:1 rate ratio was associated with phase-coupling between breathing and RRI waves. There was a characteristic pattern of coincidence of RRI minima (start of HR decrease) and early inspiration (21.3 ± 17.6% Ti) and of RRI maxima (start of HR increase) and late inspiration (79.9 ± 10.8% Ti, for an example see Figure 2A). This coupling pattern was observed in 43 ± 13% of the total recording time. This phase-coupling pattern is quite contrary to typical RSA with HR increases during inspiration and decreases during expiration. Notably, this pattern prevailed irrespective of switches in the frequencies of the rhythms. RPs were quickly restored or even maintained after occasional frequency changes (for an example see Figures 2A,B).

While the predominant phase-relation might suggest that respiration precedes the RRI rhythm, there were also events indicating a leading function of the RRI rhythm. Such events were changes in the period duration of respiratory and RRI waves that usually occurred several times during a session (see Figures 2B,C). We observed 59 transitions in period duration. In 90% of these events, the change in RRI duration preceded that of breath duration indicating that – in contrast to the conventional RSA – the RRI rhythm dominated over the respiratory rhythm.

## Discussion

The “wave-by-wave” analysis (Raßler and Kohl, 1996) characterizes each single slow wave and evaluates the phase coupling between different physiological signals. This approach allows to analyze not only the topology of waves but also the dynamics of rhythms with alternating frequency.

### Dominant Rhythms in the Respiratory and RRI Waves

One interesting result obtained in five participants with either high or low fMRI-related anxiety is the dominance of oscillations at 0.15 Hz alternating with 0.1 Hz waves during slow breathing. While the amount of reliable 0.15 Hz waves varied among subjects between 21 and 60% (mean ±*SD*: 45 ± 15%), 0.1 Hz waves occurred in 24–46% (37 ± 10%) of the total recording time.

The 0.1 Hz rhythm has often been observed in BOLD and RRI data (Pfurtscheller et al., 2017, 2018), and it is also present in respiration. A “0.15 Hz rhythm” was reported by Perlitz et al. (2004) in the reticular formation of the brain stem (medulla oblongata and pons). Main important features of this rhythm are that (i) it is broad-banded (0.12 to 0.18 Hz; mean ±*SD*: 0.15 ± 0.03 Hz), (ii) it exhibits periods of spindle waves with increasing and decreasing amplitudes, and (iii) it is phase-synchronized with respiration at 1:1 and 1:2. These 0.15 Hz oscillations also appeared in HR and blood pressure rhythms. Notably, this 0.15 Hz rhythm could have – though not necessarily – the same frequency as the breathing rhythm. The 0.1 and 0.15 Hz rhythms were found not only in respiration and RRI signals, but were also observed in ”neural BOLD oscillations” in the cingulum and amygdala (Pfurtscheller et al., 2018) and were therefore considered as central pacemaker rhythms for modulating cardiac activity.

### Respiratory Sinus Arrhythmia (RSA) and Cortical-Activity-Mediated RRI Increase

Another important result with respect to the 0.15 Hz and 0.1 Hz oscillations is that slow inspiration was accompanied by a HR deceleration (RRI increase) and expiration by a HR acceleration (RRI decrease). This coupling pattern is quite contrary to the typical RSA (Yasuma and Hayano, 2004; Rottenberg et al., 2007) with HR increases during inspiration and decreases during expiration. RSA is used as a measure of vagally mediated HRV and linked to a variety of self-regulatory processes (Porges, 1995; Rottenberg et al., 2007). It should be emphasized that the well-known pattern of inspiration-related RRI decrease (HR acceleration) and expiration-related RRI-increase reflecting RSA is not an universal phenomenon. Our data gives evidence that RSA can be “switched-off” in subjects in a fMRI situation exhibiting slow breathing waves. An example of breathing and RRI data from two subjects with low fMRI-related anxiety, one with clear RSA (*AS* = 12) and the other with cessation of RSA (*AS* = 13) is displayed in Figure 1. The in-phase behavior of unstable breathing and RRI waves during cessation of RSA and the opposite phase behavior during RSA is apparent.

There are different ways to modulate cardiac activity, either through vagus activity-mediated HR acceleration reflecting RSA or through vagus activity-mediated HR deceleration (RRI increase) after cortical activation. Examples for cortical activation in form of EEG alpha/beta power desynchronization and almost simultaneous RRI increase are the planning of a voluntary motor act (Damen and Brunia, 1987; Pfurtscheller and Lopes da Silva, 1999), the orienting reflex (Barry, 1983) and the decision making process (Pfurtscheller et al., 2013). One source of slow cortical oscillations may be intrinsic cortical excitability fluctuations (Vanhatalo et al., 2004), another could be rhythms induced through the ascending reticular activation system (Starzl et al., 1951) originating in the brainstem (Perlitz et al., 2004).

### Inspiration to Expiration Ratio (Ti/Te)

In all 5 participants with slow breathing waves, Ti was significantly longer than in both the 0.15 Hz and 0.1 Hz oscillations. The corresponding ratios (Ti/Te) were 1.5 ± 0.3 for 0.15 Hz and 2.0 ± 0.4 for 0.1 Hz oscillations. This finding contrasts to those in healthy subjects without particular emotional affection, which showed a considerably shorter Ti than Te with Ti/Te ranging between 0.65 and 0.70 for recordings over 40–70 breaths (Raßler et al., 1999, 2000; Krupnik et al., 2015). A recently published paper about relationships between state anxiety and respiration during quiet breathing in normal subjects reported a significant negative correlation between Ti, Te and anxiety indicating an increase of the respiration rate from 7 to 22 breaths/min with a predominant reduction of Ti in anxious persons (Kato et al., 2017). We would assume that a short Ti (Ti/Te < 1) is characteristic for healthy subjects without particular emotional affection and conventional RSA. A long Ti (Ti/Te > 1) may be indicative for cessation of RSA, which is often associated with a lead of the RRI rhythm (see Figures 2B,C). It can be speculated that the long Ti allows an interaction between the center of metabolic breathing in the brain stem and higher centers including the limbic system and cortical structures (Homma and Masaoka, 2008).

Summarizing, participation in fMRI scanning, an uncomfortable claustrophobic situation, can be accompanied by a slow breathing rate varying between 6 and 9 breaths/min in a minority of subjects. This slow breathing is an autonomic process, able to enhance the HRV through large RRI amplitude changes. Therewith, it activates the resources of high HRV for successful regulation of unpleasant emotions during scanning.

## Author Contributions

GP conceived the study and drafted the original manuscript. BR contributed to methodology, data processing, statistics, writing, and visualization. CA performed the ECG processing and signal-to-noise enhancement of the scanner data. AS reviewed and edited the manuscript.

## Conflict of Interest Statement

The authors declare that the research was conducted in the absence of any commercial or financial relationships that could be construed as a potential conflict of interest.

## Abbreviations

0e: start of expiration
0i: start of inspiration
AS: anxiety score
BOLD: blood-oxygenation-level-dependent
(f)MRI: (functional) magnetic resonance imaging
HF-HRV: high frequency heart rate variability
HR: heart rate
HRV: heart rate variability
LF-HRV: low frequency heart rate variability
RP: relative phase
RRI: beat-to-beat interval
RRImax: maximum within a RRI wave
RRImin: minimum within a RRI wave
RSA: respiratory sinus arrhythmia
Te: expiration time
Ti: inspiration time
Trri-de: decreasing flank of the RRI wave
Trri-in: increasing flank of the RRI wave
Trri-tot: RRI wave duration
Ttot: breath duration
